# Beneficial Effect of Voluntary Exercise on Experimental Colitis in Mice Fed a High-Fat Diet: The Role of Irisin, Adiponectin and Proinflammatory Biomarkers

**DOI:** 10.3390/nu9040410

**Published:** 2017-04-20

**Authors:** Agnieszka Irena Mazur-Bialy, Jan Bilski, Dagmara Wojcik, Bartosz Brzozowski, Marcin Surmiak, Magdalena Hubalewska-Mazgaj, Anna Chmura, Marcin Magierowski, Katarzyna Magierowska, Tomasz Mach, Tomasz Brzozowski

**Affiliations:** 1 Department of Ergonomics and Exercise Physiology, Faculty of Health Sciences, Jagiellonian University Medical College, 20 Grzegorzecka Street, 31-531 Cracow, Poland; agnieszka.mazur@uj.edu.pl (A.I.M.-B.); jan.bilski@uj.edu.pl (J.B.); afrasik@wp.pl (A.C.); 2 Department of Physiology, Faculty of Medicine, Jagiellonian University Medical College, 16 Grzegorzecka Street, 31-531 Cracow, Poland; dagmarawojcik@interia.pl (D.W.); marcin.surmiak@uj.edu.pl (M.S.); m.magierowski@uj.edu.pl (M.M.); k.jasnos@interia.pl (K.M.); 3 Gastroenterology and Hepatology Clinic, Jagiellonian University Medical College, 5 Sniadeckich Street, 31-531 Cracow, Poland; bartek.brzozowski@op.pl (B.B.); tmach@su.krakow.pl (T.M.); 4 Department of Genetic Research and Nutrigenomics, Malopolska Centre of Biotechnology, Jagiellonian University, 7A Gronostajowa Street, 30-387 Cracow, Poland; magdalena.hubalewska@uj.edu.pl

**Keywords:** voluntary exercise, colitis, diet-induced obesity, colonic blood flow, inflammation, myokines, irisin, adiponectin, tumour necrosis factor-alpha, interleukin-6

## Abstract

Inflammatory bowel diseases (IBDs) are a heterogeneous group of disorders exhibited by two major phenotypic forms: Crohn‘s disease and ulcerative colitis. Although the aetiology of IBD is unknown, several factors coming from the adipose tissue and skeletal muscles, such as cytokines, adipokines and myokines, were suggested in the pathogenesis of ulcerative colitis; however, it has not been extensively studied whether voluntary exercise can ameliorate that disorder. We explored the effect of moderate exercise (i.e., voluntary wheel running) on the disease activity index (DAI), colonic blood flow (CBF), plasma irisin and adiponectin levels and real-time PCR expression of proinflammatory markers in mesenteric fat in mice with 2,4,6-trinitrobenzenesulfonic acid (TNBS) colitis fed a high-fat diet (HFD) compared to those on a standard chow diet (SD). Macroscopic and microscopic colitis in sedentary SD mice was accompanied by a significant fall in CBF, some increase in colonic tissue weight and a significant increase in the plasma levels of tumour necrosis factor-alpha (TNF-α), IL-6, monocyte chemotactic protein 1 (MCP-1) and IL-13 (*p* < 0.05). In sedentary HFD mice, colonic lesions were aggravated, colonic tissue weight increased and the plasma TNF-α, IL-6, MCP-1, IL-1β and leptin levels significantly increased. Simultaneously, a significant decrease in the plasma irisin and adiponectin levels was observed in comparison with SD mice (*p* < 0.05). Exercise significantly decreased macroscopic and microscopic colitis, substantially increased CBF and attenuated the plasma TNF-α, IL-6, MCP-1, IL-1β and leptin levels while raising the plasma irisin and the plasma and WAT concentrations of adiponectin in HFD mice (*p* < 0.05). We conclude that: (1) experimental colitis is exacerbated in HFD mice, possibly due to a fall in colonic microcirculation and an increase in the plasma and mesenteric fat content of proinflammatory biomarkers; and (2) voluntary physical activity can attenuate the severity of colonic damage in mice fed a HFD through the release of protective irisin and restoration of plasma adiponectin.

## 1. Introduction

Inflammatory bowel diseases (IBDs) pertain to a group of disorders of inflammatory conditions of the colon and small intestine that display two major phenotypic forms: Crohn’s disease (CD) and ulcerative colitis (UC), both characterized by a cyclical nature alternating between active and quiescent states [1]. Although progress has been made in the understanding of IBDs, their aetiology is unknown; however, an aberrance in cytokine secretion seems to be involved in the pathogenesis of IBDs [1]. Even though the majority of CD patients are underweight, their ratios of intraabdominal fat to total abdominal fat are far greater than those in control groups, as assessed by magnetic resonance imaging (MRI) [2]. In CD, the hypertrophied mesenteric white adipose tissue (mWAT) could be a major contributor to the increase in the circulating proinflammatory cytokines and play a role in the pathogenesis and disease activity [2,3,4,5]. Diet-induced obesity (DIO) obtained by feeding rodents on high-fat diets is the most common model reported in the literature because DIO mimics the human characteristics of obesity and leads to increased mesenteric fat deposition, colonic inflammation and deterioration in experimental colitis [6,7,8,9].

Increased aerobic activity, designed specifically for IBD patients, has been recommended for obtaining good overall health, reversing muscle weakness and improving bone mineral density [10]. A potential mechanism to promote the beneficial effects of exercise in IBD is the secretion of muscle-derived peptides, called ‘myokines,’ from active skeletal muscles [5,11]. A recent discovery of these muscle hormones and their ability to modify adipose tissue metabolism in mice and humans constitutes significant progress in the understanding of muscle–fat crosstalk and metabolic disorders [12].

Experimental exercise models in mice involve either voluntary or forced physical activity. Forced model patterns of activity are, however, far removed from normal mouse behaviour and experiments are usually performed during daylight hours, contrary to the murine diurnal pattern of nocturnal activity [13]. An alternative is voluntary wheel running, which occurs in a non-stressed environment without disruption to the normal murine diurnal rhythm, and assessment of the training response over time [14]. It was observed that forced treadmill exercise exacerbated inflammation, while voluntary exercise was protective in experimental colitis [15]. It is interesting that intensive exercise in humans, such as long-distance running, could lead to ‘runner’s ischaemic colitis’ involving bloody diarrhoea, fatigue and fever [16].

Therefore, the major aim of the present study was to determine the effect of voluntary exercise on experimental colitis caused by intrarectal administration of TNBS in mice fed a normal standard diet (SD) or high-fat diet (HFD). We aimed to test the hypothesis that a HFD augments the severity of experimental colitis in mice and to check whether such an effect could be prevented, even partly, by prior voluntary exercise, possibly by modifying the muscle–adipose tissue crosstalk, myokine release and by attenuation of expression and release of proinflammatory biomarkers.

## 2. Materials and Methods

### 2.1. Animals and Diets

Animal studies were carried out on male C57BL/6J mice kept in a pathogen-free room with free access to water and food and adapted to laboratory conditions and to the 12 h/12 h day/night cycles. The mice were fed ad libitum for 12 weeks either on regular chow pellets as a standard diet (SD): diet C 1000; Altronim, Lage, Germany, or on an obesity-inducing diet with 70% of energy obtained from fat (42% fat): diet C 1090-70; Altromin, Lage, Germany, as a high-fat diet (HFD) containing cholesterol at the concentration of 200 mg/kg. Following is a link to the detailed constituents of both the SD and HFD: (http://www.altromin.com/products/special-and-experimental-diets/data-specifications-of-special-diets/#kontroll).

The energy values for both diets are: C 1000 (control): 3518.05 kcal/kg and C 1090-70 Obesity: 5495.855 kcal/kg. The standard diet C 1000 was not contaminated with phytoestrogens from soy, as they might interfere with the differences between the results obtained with a high-fat diet (information from the manufacturer).

The study was approved by a local Ethics Committee at the Jagiellonian University Medical College in Cracow, Poland (No. 158/2013) and was run in accordance with the Declaration of Helsinki.

### 2.2. Experimental Design

**Figure 1:**
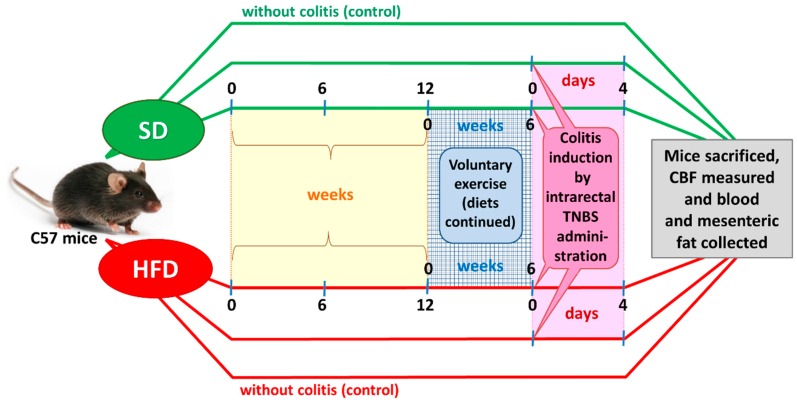
A flow chart presenting the experimental protocol and time duration of this study.

After an adaptation period, the SD and DIO mice were randomized into four experimental series, each consisting of 8–15 animals per group: (1) sedentary mice kept on SD; (2) mice maintained on SD and subjected to voluntary physical activity; (3) sedentary mice fed a HFD; and (4) mice fed a HFD and subjected to voluntary exercise. After 12 weeks of HFD (C 1090-70 Obesity diet) and SD (C 1000) feeding (Figure 1), two groups of animals were subjected to voluntary wheel running for us to assess the effect of physical performance and endurance on the course of colitis. The remaining groups were considered sedentary animals without exercise. For the endurance test, the mice housed individually were allowed to run freely in open surface wheels placed outside their standard mouse cages. The voluntary-exercised animals were housed in cages equipped with a running wheel assembly (Activity Wheel and Living Chamber; Lafayette Instrument Company, Lafayette, IN, USA) connected to a switch that counted each revolution of the wheel and recorded it daily for a period of six weeks. During the experiment, the sedentary and voluntary-exercising mice were still maintained on the C 1000 and C 1090-70 Obesity diets, respectively. Rotations were electronically transmitted to an USB hub, so that the frequency and running rate could be captured by software dedicated to data storage and analysis for variable time periods.

After six weeks of wheel exercise, colitis was induced in both groups of the sedentary and exercising mice by intracolonic administration of 2,4,6-trinitrobenzenesulfonic acid (TNBS), as described elsewhere [17]. Energy intake (kilocalories per day) was measured daily, while body weight was monitored once weekly during the period of the present study.

### 2.3. Induction of Colitis

The animals were anaesthetized with isoflurane and experimental colitis in the randomly assigned lean SD and DIO mice was induced by intracolonic administration of TNBS (Sigma, Slough, UK) at a dose of 100 μg/g in 40% ethanol. For this purpose, a 100 μL volume dissolved in a 40% solution of ethanol or an equal volume of a 0.9% saline solution was instilled transrectally on day 0. The animals in the control group were administered with 40% ethanol at a volume of 0.25 mL per mouse, which corresponded with the volume given to the mice administered with TNBS. Following the induction of colitis, the animals were housed individually, and their daily food intake and body weight were monitored. Exercise sessions were stopped with the induction of colitis with TNBS. At day 4 post-induction, the animals were weighed and anaesthetized to determine CBF using the H_2_-gas clearance technique. The abdominal cavity was opened and after separation of the colon, CBF was measured in the areas of colonic mucosa unaffected by inflammatory lesions. The disease activity index (DAI) and rectal bleeding scores were calculated using a modification of a previously published compounded clinical score. In brief, the DAI comprised the scoring for diarrhoea and lethargy (0–3) and rectal bleeding assessment involved a visual inspection of blood in faeces and the perianal area (0–4) [17]. Blood was drawn from the vena cava and the plasma concentrations of TNF-α, MCP-1, IL-6, IL-13 and irisin along with the fat concentration of TNF-α, MCP-1, IL-6, adiponectin and leptin were assessed.

At the end of these experiments, the total mesenteric white adipose tissue and the entire colon were removed, then they were isolated from the surrounding tissue, rinsed, weighed and processed for gross determination of intestinal damage and histology examination. Subsequently, fragments of the colon (2 mm × 10 mm) with colonic lesions were sampled, fixed with formaldehyde, embedded in paraffin and routinely stained with haematoxylin and eosin for histological assessment. The presence and intensity of histological changes were evaluated for the following criteria: the presence, area and depth of ulceration, the presence and intensity of inflammatory infiltrations, ulcerations and fibrosis. The colons were graded with a compounded histological score covering the extent of: (1) crypt damage; (2) regeneration; (3) metaplasia/hyperplasia; (4) vascular changes in the lamina propria; (5) submucosal changes; and (6) the presence of inflammatory infiltrates. The sections were graded from 0 to 4 for each of the above categories and the data were analysed as a normalized compounded score [18].

### 2.4. Quantitative Real-Time PCR Assay

Quantitative real-time polymerase chain reaction (qRT-PCR) was used to determine gene expression in mesenteric adipose tissue samples. For that method, the total RNA from adipose tissue was isolated using an RNeasy Plus Mini Kit (Qiagen, Hilden, Germany). The concentration and quality of RNA were measured using a NanoDrop 2000 spectrophotometer (Thermo Scientific, Wilmington, DE, USA). RNA was reverse-transcribed using a High Capacity RNA-to-cDNA Kit (Applied Biosystems, Foster City, CA, USA). Real-time polymerase chain reaction (PCR) was performed using a StepOne Plus system (Applied Biosystems, Foster City, CA, USA) and applying a TaqMan Gene Expression Master Mix (Applied Biosystems) and TaqMan Gene Expression Assays (Applied Biosystems) for TNF-α (Assay ID: Mm00443260_g1), IL-6 (Assay ID: Mn00446190_m1), MCP-1 (Assay ID: Mm00441242_m1), adiponectin (Assay ID: Mm00456425_m1) and leptin (Assay ID: Mm00434759_m1). PCR amplification was performed under the following conditions: denaturation at 95 °C for 10 min followed by 40 cycles at 95 °C for 15 s and then by primer hybridization and extension at 60 °C for 1 min. Endogenous glyceraldehyde phosphate dehydrogenase (GAPDH) expression was used as housekeeping control (Assay ID: Mm99999915_g1). The relative expression of each gene (RQ) was calculated using the 2-^ΔΔ*C*t^ method according to Livak and Schmittgen [19].

### 2.5. Luminex Microbeads Fluorescent Assays

Determination of serum levels of cytokines (IL-6, IL-13, MCP-1, TNF-α, IL-17, KC, IL-1α and IL-4) was performed using Luminex microbeads fluorescent assays (Novex: Mouse Cytokine Magnetic 20-Plex, Life Technologies, Carlsbad, CA, USA) and a MAGPIX fluorescent-based detection system (Luminex Corp., Austin, TX, USA). Results were calculated based on the calibration curves in pg/mL.

### 2.6. Statistical Analysis

Results are expressed as means ± S.E.M. The data was processed by the statistical analysis software SPSS version 16.0 (SPSS Inc., Chicago, IL, USA). Statistical analysis was performed using either Student’s *t*-test to compare two groups or a two-way ANOVA test with Tukey post hoc test, if more than two experimental groups were compared. Differences of *p* < 0.05 were considered significant.

## 3. Results

### 3.1. Effects of High-Fat Diet on Energy Intake, Running Distance, Body Weight and Visceral Adiposity in Sedentary Mice and Those Subjected to Voluntary Exercise

**Figure 2:**
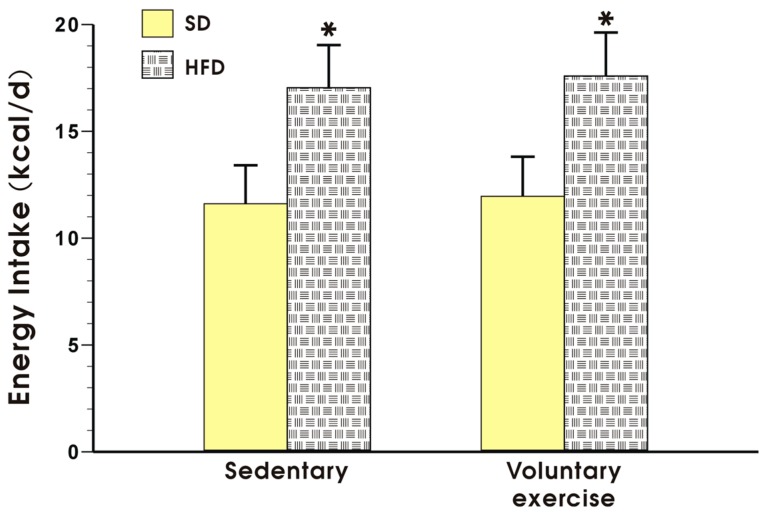
Energy intake in the mice fed a SD or HFD with or without voluntary exercise. Results are mean ± standard error of the mean S.E.M. of eight animals per each group. An asterisk indicates a significant change as compared to the respective values in the sedentary mice fed a SD with or without voluntary exercise (*p* < 0.05, *t*-test).

Figure 2 shows the energy intake expressed in kcal/day in the sedentary mice fed a SD and those fed a HFD, with or without voluntary exercise. Energy intake was not significantly different between the sedentary and the exercising mice fed a SD but it was significantly increased in the animals fed a HFD as compared to the energy value obtained in the mice fed a SD (*p* < 0.05, Figure 2).

**Figure 3:**
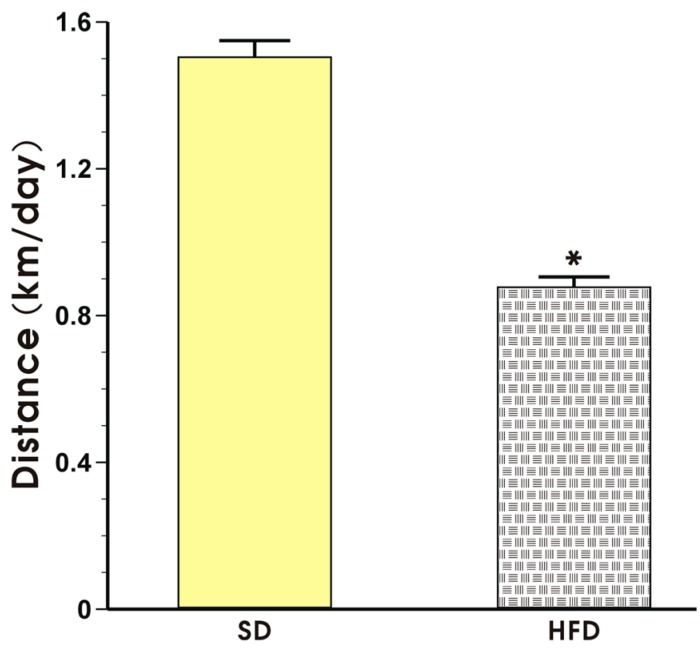
The running distance in the mice fed a SD or HFD and subjected to voluntary exercise. Note that the animals were initially fed a SD and HFD over the period of 12 weeks and subsequently subjected to voluntary exercise for a period of six weeks. Results are mean ± S.E.M. of eight animals per each group. An asterisk indicates a significant change as compared to the respective values in sedentary exercising mice fed a SD (*p* < 0.05, *t*-test).

The effect of voluntary exercise on the running distance in the mice fed a SD or HFD is presented in Figure 3. The mice in both groups exhibited a regular pattern of nocturnal running but the running distance expressed in km per day in the mice fed a HFD was significantly shorter than that recorded in the mice fed a SD (*p* < 0.05, Figure 3).

**Figure 4:**
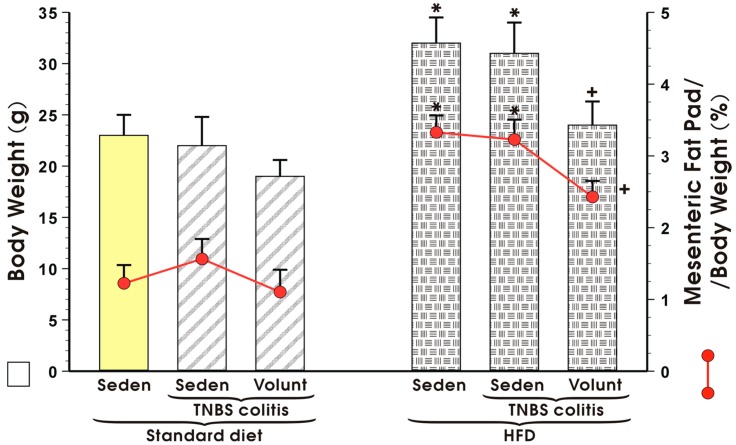
Body weight in grams and mesenteric fat pad per body weight (%) in sedentary (Seden) or voluntary exercising (Volunt) mice with or without TNBS-induced colitis fed a standard diet (SD) or a high-fat diet (HFD). Results are mean ± S.E.M. of eight animals per each group. An asterisk indicates a significant change as compared to the respective values in the sedentary mice with or without colitis fed a HFD (*p* < 0.05, ANOVA). A cross indicates a significant change as compared to the respective values in the sedentary mice with colitis fed a HFD (*p* < 0.05, ANOVA).

Figure 4 illustrates animal body weight and the weight of mesenteric fat depots expressed as a percentage in the sedentary or exercising mice with or without TNBS colitis fed a standard diet (SD) or high-fat diet (HFD). Voluntary exercise failed to influence the body weight or mesenteric fat pads but tended to decrease these values, although not significantly, in the sedentary SD mice with colitis (Figure 4). The body weight in the HFD mice without colitis was significantly increased as compared to the sedentary animals fed a SD and the relative weight of the mesenteric fat depots expressed as percentage was also significantly increased in the HFD mice as compared to the SD mice (*p* < 0.05) (Figure 4). In contrast, voluntary exercise significantly reduced the body weight and mesenteric fat depots in the mice with colitis fed a HFD (*p* < 0.05, Figure 4).

### 3.2. The Effect of Voluntary Exercise on the Macroscopic and Microscopic Appearance of TNBS-Induced Colitis in Mice Fed a SD or HFD

**Figure 5:**
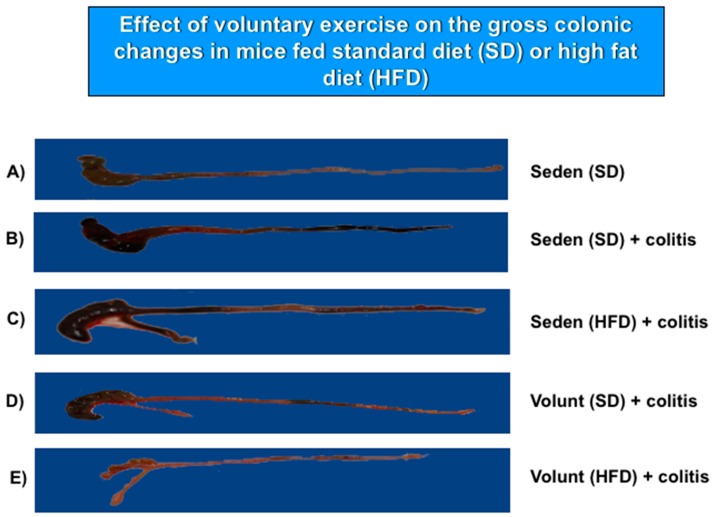
The representative gross appearance of the colon obtained from: (**A**) a sedentary mouse without colitis fed a standard diet (SD); (**B**) a sedentary mouse with TNBS colitis fed a standard diet (SD); (**C**) a sedentary mouse with TNBS colitis fed a HFD without voluntary exercise; and (**E**) a mouse with TNBS colitis fed a HFD, subjected to voluntary exercise and sacrificed at day 4 after the induction of colitis. The photomicrographs represent: (**A**) a normal colon from a sedentary mouse without colitis fed a SD: normal macroscopic appearance of the colonic mucosa; (**B**) a colon from a sedentary mouse with colitis fed a SD: haemorrhagic lesions and a bloody effusion; (**C**) a colon from a sedentary mouse with colitis fed a HFD: more severe haemorrhagic lesions with a bloody effusion involving the entire intestine; (**D**) a colon from a sedentary mouse fed a SD with voluntary exercise: a less severe intestinal damage and bleeding; (**E**) a colon from a mouse fed a HFD with voluntary exercise: marked improvement in colitis as manifested by a reduction in bleeding, haemorrhagic lesions and macroscopic signs of mucosal injury.

**Figure 6:**
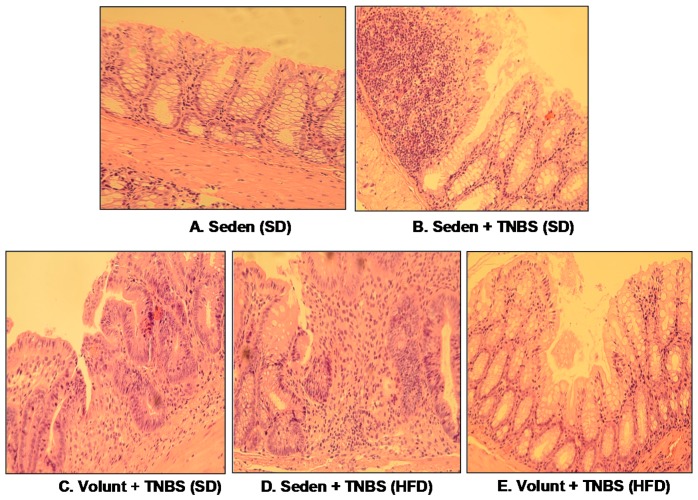
The representative microscopic appearance of the colonic mucosa in: a sedentary mouse without TNBS colitis fed a SD (**A**); a sedentary mouse with TNBS colitis fed a SD (**B**); one with colitis fed a SD and subjected to voluntary exercise (**C**); a sedentary mouse with colitis fed a HFD (**D**); a mouse with colitis fed a HFD and subjected to voluntary exercise (**E**). The colonic mucosa of the sedentary mouse (**A**) shows normal crypt architecture but inflammatory exudates and mucosal damage are observed in the sedentary mouse fed a SD and administered with TNBS (**B**); that inflammatory reaction is less intense and the mucosal damage is attenuated in the colonic mucosa of the TNBS mouse fed a SD and subjected to voluntary exercise (**C**). Colonic inflammation, neutrophil infiltration and a loss in the mucosal architecture are worsened in the sedentary mouse with colitis fed a HFD (**D**). In contrast, the microscopic appearance of colonic mucosa in the TNBS mouse fed a HFD and subjected to voluntary exercise showed a less severe inflammation and neutrophil infiltration, and attenuation of colonic damage with signs of regeneration as compared to the sedentary mouse with HFD (**E**).

Figure 5 shows the representative gross macroscopic appearance of the colon from: a sedentary control mouse without colitis fed a SD (A); a sedentary mouse with TNBS colitis fed a SD (B); a mouse with TNBS colitis fed a HFD (C); a mouse with TNBS colitis fed on SD and subjected to voluntary exercise (D); and a mouse with TNBS colitis fed a HFD and subjected to voluntary exercise (E). The colon from the sedentary mouse fed a SD was normal but intrarectal administration of TNBS in the sedentary mice fed a SD revealed clearly visible bloody effusions reflecting mucosal intestinal injury (Figure 5A vs. Figure 5B). These macroscopic changes were more severe in the sedentary mice with colitis fed a HFD as compared to those fed a SD without exercise (Figure 5B,C). In contrast, a reduction in the inflammatory reaction and bloody effusion were observed in the mouse fed either a SD or HFD and subjected to voluntary exercise as compared to the sedentary mouse fed a SD or HFD (Figure 5D vs. Figure 5B,E vs. Figure 5C). In histology, severe mucosal injury characterised by necrosis of the epithelium and focal lesions of the colonic mucosa were observed in the sedentary mouse with TNBS colitis fed a SD as compared to that observed in the sedentary mouse without colitis fed a SD (Figure 6B vs. Figure 6A). These microscopic alternations in the colonic mucosa were augmented in the sedentary mouse fed a HFD and administered with TNBS, indicating that the course of colitis in these animals was worsened as compared to the mice fed a SD (Figure 6D vs. Figure 6B). As shown in Figure 6, the microscopic damage observed in the TNBS colitis mice fed either a SD or HFD was attenuated when the mice were subjected to voluntary exercise, as compared to the non-exercising animals and as reflected by a less severe damage, moderate neutrophil infiltration and distinguished signs for mucosal regeneration and healing (Figure 6C,E vs. Figure 6B,D).

**Figure 7:**
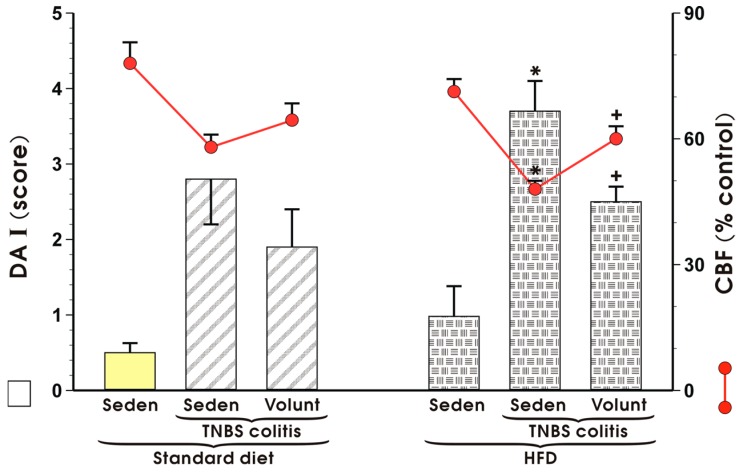
The effect of exercise on the disease activity index (DAI) and alterations in colonic blood flow (CBF) in sedentary (Seden) animals with TNBS-induced colitis fed a standard diet (SD) or a high-fat diet (HFD), with or without voluntary exercise (Volunt). Results are mean ± S.E.M. of eight animals per each group. An asterisk indicates a significant change as compared to the respective values in the colitis mice fed a SD (*p* < 0.05, *t*-test). A cross indicates a significant change as compared to the respective values obtained in the animals fed a HFD but not subjected to exercise (*p* < 0.05, *t*-test).

Figure 7 shows changes in the mucosal DAI and alterations in CBF in sedentary mice with TNBS colitis fed a SD and those fed a HFD with or without voluntary exercise. DAI was significantly increased and CBF was substantially reduced in the HFD mice with colitis as compared to the respective values recorded in those maintained on a standard diet (*p* < 0.05) (Figure 7). Increase in DAI and fall in CBF observed in the mice fed a HFD were reverted when the mice fed a HFD were subjected to voluntary exercise as compared to those maintained under sedentary conditions and fed a HFD (*p* < 0.05) (Figure 7).

### 3.3. The Effects of SD and HFD on the Plasma Concentration of Cytokines, Myokine Irisin, Leptin and Adiponectin in TNBS Colitis Mice with or without Voluntary Exercise

**Figure 8:**
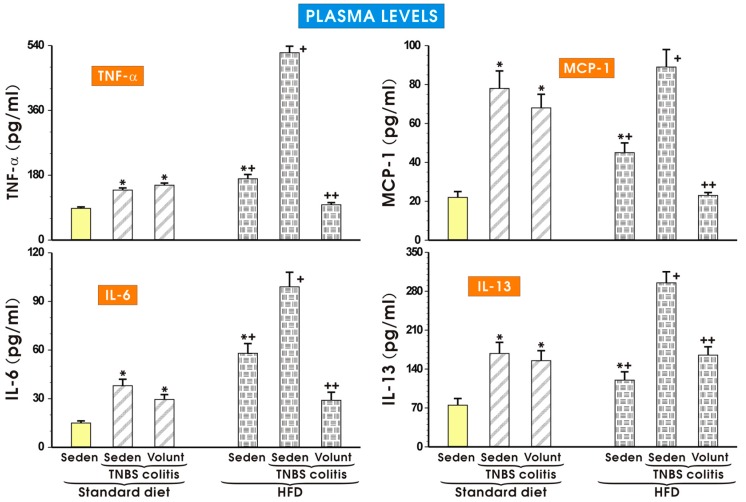
The plasma levels of TNF-α, MCP-1, IL-6 and IL-13 in sedentary mice with colitis fed a standard diet (SD) or high-fat diet (HFD) with or without voluntary exercise (Volunt). Please note the inhibitory effect of voluntary exercise on the proinflammatory biomarkers observed in the HFD mice with colitis. Results are mean ± S.E.M. of eight mice per each group. An asterisk indicates a significant difference as compared to the control groups of animals without colitis (*p* < 0.05, ANOVA). An asterisk and a cross indicate a significant difference as compared to the values obtained in rats fed a SD (*p* < 0.05, ANOVA). A cross indicates a significant difference as compared to the values obtained in the sedentary mice without colitis fed a HFD (*p* < 0.05, ANOVA). A double cross indicates a significant change as compared to the HFD mice without voluntary exercise (*p* < 0.05, ANOVA).

The results of the plasma concentrations of TNF-α, MCP-1, IL-6 and IL-13 in the sedentary mice kept on SD and those fed a HFD and exposed to TNBS with or without voluntary exercise are presented in Figure 8. In the sedentary group fed a SD, the plasma TNF-α, MCP-1, IL-6 and IL-13 were negligible but in the mice with colitis, a significant increase in the plasma levels of these cytokines was observed (*p* < 0.05) (Figure 8). These effects in the sedentary TNBS mice fed a SD were not significantly affected by voluntary exercise. A significant increase in the plasma levels of TNF-α, MCP-1, IL-6 and IL-13 was observed in the sedentary mice fed a HFD and these changes were further significantly elevated in the HFD mice exposed to TNBS (*p* < 0.05). In contrast, such an elevation in the plasma levels of TNF-α, MCP-1, IL-6 and IL-13 was significantly attenuated in the HFD mice subjected to voluntary exercise prior to the induction of colitis (*p* < 0.05) (Figure 8).

**Figure 9:**
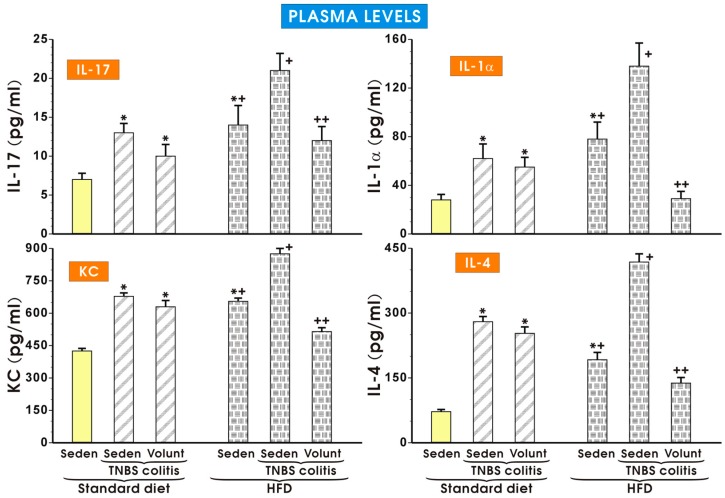
The effect of exercise on the plasma levels of IL-17, IL-1α, KC and IL-4 in the sedentary mice with colitis fed a standard diet (SD) or those with colitis fed a high-fat diet (HFD), with or without voluntary exercise. Please note the inhibitory effect of voluntary exercise on the proinflammatory biomarkers observed in the HFD mice with colitis. Results are mean ± S.E.M. of eight mice per group. An asterisk indicates a significant difference as compared to the control group of sedentary animals without colitis (*p* < 0.05, ANOVA). An asterisk and cross indicate a significant difference as compared to the values obtained in the sedentary mice without colitis fed a SD (*p* < 0.05, ANOVA). A cross indicates a significant difference as compared to the values obtained in the sedentary mice without colitis fed a HFD (*p* < 0.05, ANOVA). A double cross indicates a significant change as compared to the values obtained in the colitis mice fed a HFD without voluntary exercise (*p* < 0.05, ANOVA).

As shown in Figure 9, the plasma concentrations of IL-17, KC, IL-1a and IL-4 were significantly increased in the sedentary mice with colitis fed a SD as compared to the respective values obtained in the sedentary mice without colitis fed a SD (*p* < 0.05). The plasma levels of IL-17, KC, IL-1a and IL-4 tended to decrease in the colitis mice fed a SD and subjected to voluntary exercise as compared to the non-exercising mice (*p* < 0.05) but that change was insignificant. Similarly to the data presented in Figure 8, the plasma levels of IL-17, KC, IL-1a and IL-4 were significantly increased in the animals without colitis fed a HFD as compared to the respective values in the sedentary mice fed a SD. The concentrations of those cytokines were further significantly increased in those fed a HFD and administered with TNBS (*p* < 0.05) (Figure 9). Voluntary exercise in the group of mice with colitis fed a HFD caused a significant reduction in the plasma levels of these cytokines as compared to the respective values recorded in the non-exercising sedentary mice with colitis fed a HFD (*p* < 0.05) (Figure 9).

**Figure 10:**
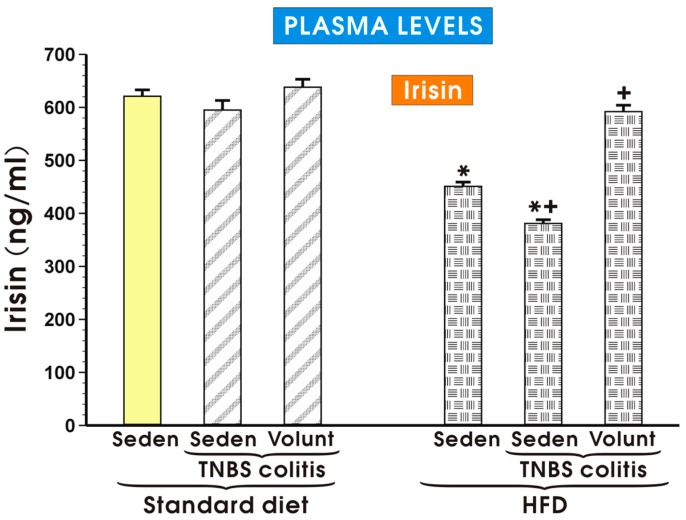
The plasma irisin levels in the sedentary mice (Seden) with or without TNBS colitis fed a standard diet (SD) or a high-fat diet (HFD) and subjected or not subjected to voluntary exercise (Volunt). Please note that the plasma irisin concentration was significantly diminished in the sedentary mice fed a HFD and that effect was reversed in the HFD mice subjected to voluntary exercise. Results are mean ± S.E.M. of eight animals per each experimental group. An asterisk indicates a significant change as compared to the respective values in the sedentary mice without colitis fed a SD (*p* < 0.05, *t*-test). An asterisk and a cross indicate a significant change as compared to the respective values in the colitis animals fed a SD (*p* < 0.05, *t*-test). A cross indicates a significant change as compared to the value obtained in the sedentary animals with colitis fed a HFD without voluntary exercise (*p* < 0.05, *t*-test).

The plasma myokine irisin concentrations in the sedentary mice and in the HFD mice with colitis with or without voluntary exercise are presented in Figure 10. The plasma irisin concentrations remained unchanged in the sedentary mice with colitis fed a SD as compared to the levels detected in the control sedentary mice without colitis fed a SD. Voluntary exercise tended to increase the plasma irisin levels in the sedentary mice with colitis fed a SD but that change failed to reach statistical significance (Figure 10). The plasma irisin levels were significantly decreased in the sedentary mice with or without colitis fed a HFD (*p* < 0.05). In the colitis mice fed a HFD and subjected to voluntary exercise, a significant increase in the plasma irisin levels was observed as compared to the respective values of irisin recorded in the HFD animals with colitis kept without voluntary exercise (Figure 10).

**Figure 11:**
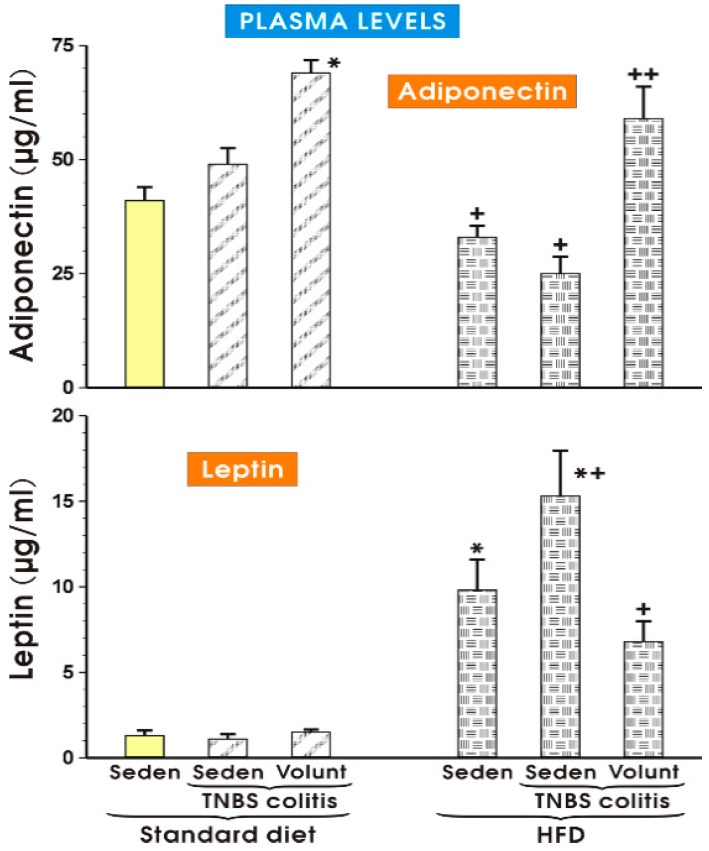
The plasma adiponectin and leptin levels in the sedentary mice with or without TNBS colitis fed a standard diet (SD) or a high-fat diet (HFD) subjected or not subjected to voluntary exercise (Volunt). Results are mean ± S.E.M. of eight animals per each experimental group. An asterisk indicates a significant change as compared to the respective values in the sedentary mice without colitis fed a SD (*p* < 0.05, *t*-test). An asterisk and a cross indicate a significant change as compared to the respective values in the TNBS colitis mice fed a SD (*p* < 0.05, *t*-test). A cross indicates a significant change as compared to the values obtained in the sedentary animals with colitis fed a HFD without voluntary exercise (*p* < 0.05, *t*-test).

The results of plasma adiponectin and leptin levels determined in the sedentary or exercising mice fed a SD and those with or without colitis fed a HFD are presented in Figure 11. The concentration of adiponectin in the sedentary mice with colitis fed a SD tended to increase more than that recorded in the SD animals without colitis, but the increase was insignificant (Figure 11). In the animals fed a SD with voluntary exercise, a significant rise in the plasma adiponectin was observed as compared to the respective value of that adipokine in the sedentary animals fed a SD (*p* < 0.05). In contrast, the plasma levels of adiponectin were significantly decreased in the sedentary mice fed a HFD with or without colitis as compared to the concentrations of adiponectin in the sedentary mice with or without colitis fed a SD. That effect in the HFD animals with colitis was reversed when the mice fed a HFD were subjected to voluntary exercise (*p* < 0.05) (Figure 11). The plasma leptin concentrations did not differ significantly in the sedentary colitis mice fed a SD or in those fed a SD and subjected to exercise as compared to the animals without colitis fed a SD (Figure 11). In contrast, the plasma levels of leptin were significantly raised in the sedentary mice without colitis fed a HFD, while a further significant increase in the plasma levels of leptin was observed in those with colitis fed a HFD (*p* < 0.05) (Figure 11). The plasma leptin levels were significantly decreased in the mice with TNBS colitis subjected to voluntary exercise as compared to the values obtained in the group of TNBS mice fed a HFD without voluntary exercise (*p* < 0.05) (Figure 11).

### 3.4. The Effects of Standard Diet and HFD on the Expression of Proinflammatory Biomarkers in White Adipose Tissue of Mice with TNBS Colitis

**Figure 12:**
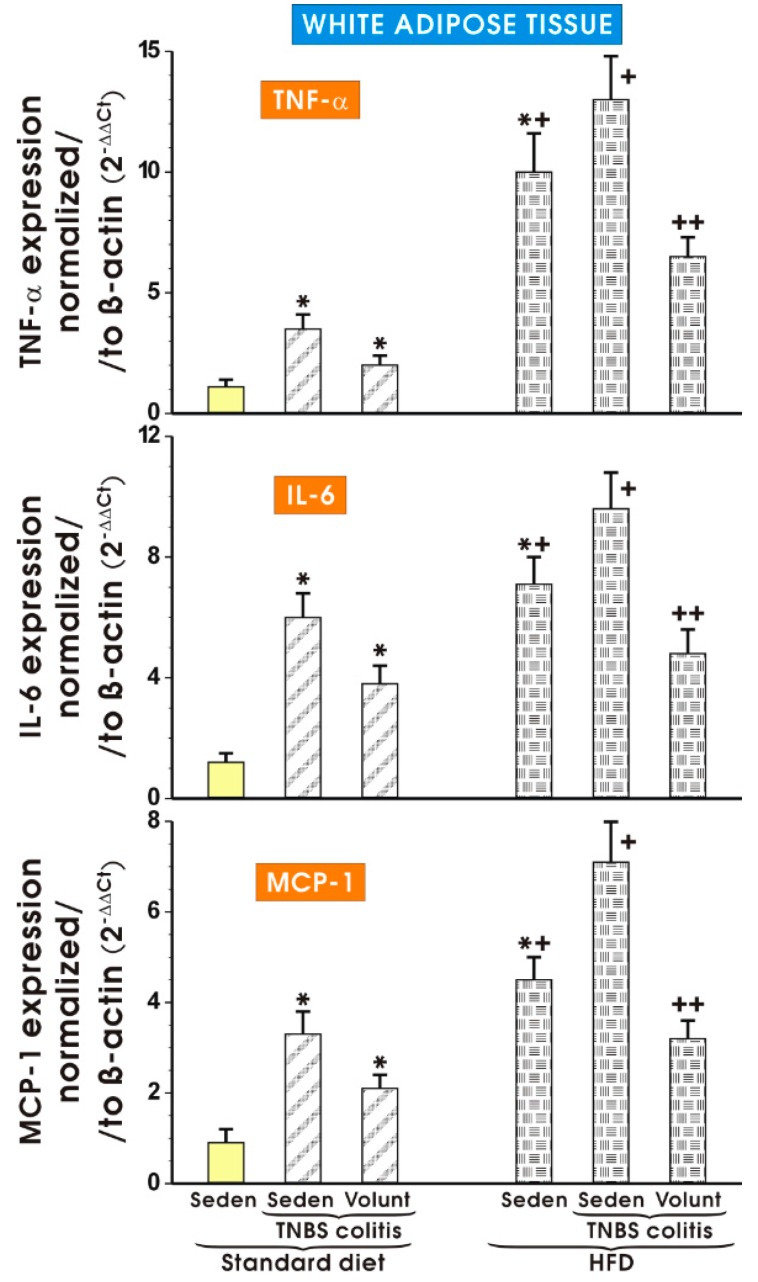
The effect of exercise on the real-time PCR expression of mRNA for TNF-α, IL-6 and MCP-1 in the mesenteric fat of sedentary mice with colitis fed a SD or HFD with or without voluntary exercise. Results are mean ± S.E.M. of eight animals per each experimental group. An asterisk indicates a significant change (*p* < 0.05, ANOVA) as compared to the respective values in the sedentary mice fed a SD. An asterisk and a cross indicate a significant change (*p* < 0.05, ANOVA) as compared to the respective values in the sedentary animals with colitis fed a SD. A cross indicates a significant change (*p* < 0.05, ANOVA) as compared to the values obtained in the sedentary colitis mice fed a HFD without exercise. A double cross indicates a significant change (*p* < 0.05, ANOVA) as compared to the values obtained in the group of sedentary HFD animals with colitis not subjected to voluntary exercise.

Figure 12 shows the results of mRNA expression for TNF-α, IL-6 and MCP-1 assessed by qPCR in the mesenteric white tissue of sedentary mice with colitis fed a SD as well as those with colitis fed a HFD with or without voluntary exercise. In the sedentary mice fed a SD, a significant increase in the expression of TNF-α, IL-6 and MCP-1 was observed (*p* < 0.05). Such an increase in the expression of mRNA for those proinflammatory biomarkers was not significantly altered by voluntary exercise (Figure 12). The expression of mRNA for TNF-α, IL-6 and MCP-1 was significantly increased in the sedentary mice fed a HFD as compared to the sedentary mice with colitis fed a SD. A further significant increase in the expression of TNF-α, IL-6 and MCP-1 mRNA was recorded in the mice with colitis fed a HFD as compared to the sedentary mice without colitis fed a HFD (*p* < 0.05) (Figure 12). The expression of mRNA for TNF-α, IL-6 and MCP-1 was significantly decreased in the mesenteric white tissue of the mice with TNBS colitis subjected to voluntary exercise as compared to the group maintained without voluntary exercise (*p* < 0.05) (Figure 8).

**Figure 13:**
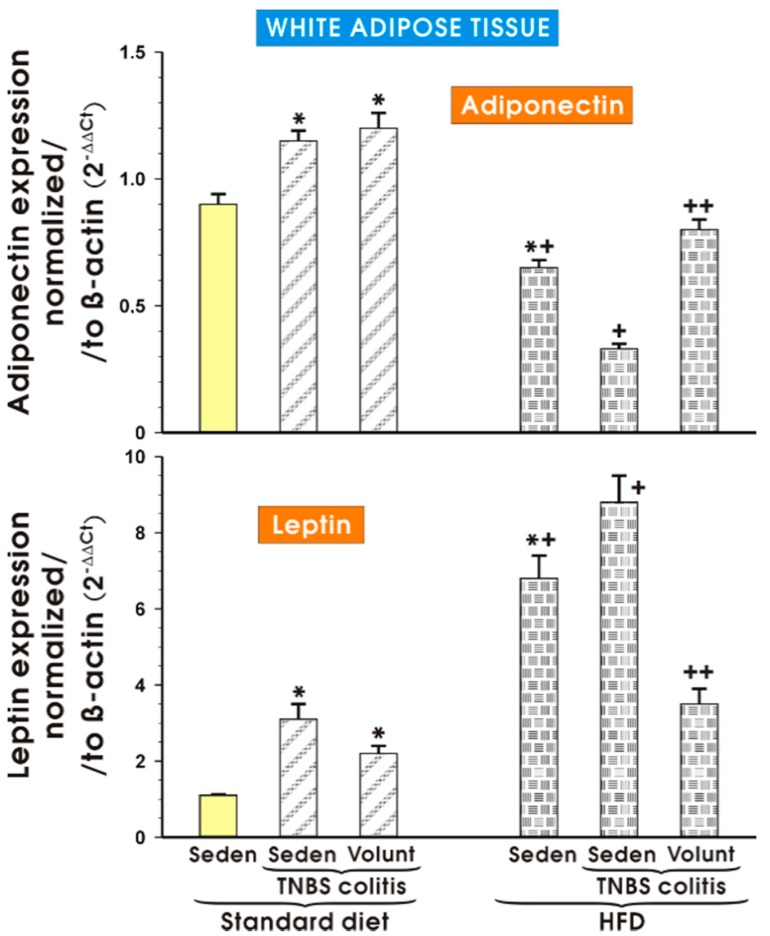
The effect of exercise on the real-time PCR expression of adiponectin and leptin in the mesenteric fat of sedentary mice with or without TNBS colitis fed a SD or HFD and subjected or not subjected to voluntary exercise. Results are mean ± S.E.M. of eight animals per each experimental group. An asterisk indicates a significant change as compared to the respective values in the sedentary mice fed a SD (*p* < 0.05, ANOVA). An asterisk and a cross indicate a significant change as compared to the respective values in the animals with colitis fed a SD (*p* < 0.05, ANOVA). A cross indicates a significant change as compared to the values obtained in the sedentary mice fed a HFD without exercise (*p* < 0.05, ANOVA). A double cross indicates a significant change as compared to the values obtained in the sedentary HFD animals with colitis not subjected to voluntary exercise (*p* < 0.05, ANOVA).

Figure 13 shows the results of qPCR expression of the adiponectin and leptin mRNAs assessed in the mesenteric white tissue of the sedentary mice with or without colitis fed a SD and those fed a HFD with or without voluntary exercise. In the sedentary mice fed a SD, a significant increase in the expression of adiponectin and leptin was observed (*p* < 0.05) and the increase in the expression of these proinflammatory biomarkers was not significantly altered by voluntary exercise (Figure 13). The expression of mRNA for adiponectin was significantly decreased in the sedentary mice fed a HFD as compared to the sedentary mice with or without colitis fed a SD (*p* < 0.05). In contrast, the expression of mRNA for leptin was significantly increased in the sedentary mice fed a HFD as compared to the sedentary mice with or without colitis fed a SD (*p* < 0.05). A further significant rise in the expression of leptin mRNA was recorded in the mice with colitis fed a HFD as compared to the values achieved in the sedentary mice without colitis fed a HFD (*p* < 0.05) (Figure 13). The expression of adiponectin mRNA, which was significantly decreased in the sedentary mice without colitis fed a HFD as compared to the sedentary mice without colitis fed a SD (*p* < 0.05), was further significantly decreased in the mice with TNBS colitis fed a HFD (*p* < 0.05) (Figure 13). The expression of mRNA for adiponectin was significantly increased but the expression of leptin mRNA was significantly decreased in the mesenteric white tissue of the mice with TNBS colitis subjected to voluntary exercise as compared to the group maintained without voluntary exercise (*p* < 0.05) (Figure 13).

## 4. Discussion

The present study demonstrated that experimental colitis was exacerbated in the mice fed a HFD and that such an effect was associated with profound changes in the plasma levels of proinflammatory biomarkers and the expression of proinflammatory factors in the mesenteric WAT. Furthermore, a marked fall in the colonic microcirculation followed by an increase in the DAI index and plasma proinflammatory markers as well as a decrease in the potentially protective factors such as FNDC5/irisin and adiponectin can likely be attributed to the effects of increased mesenteric adiposity observed in the HFD mice with colitis. Our major observation in this study was that voluntary physical activity, despite a shorter running distance for the HFD mice than those fed a SD, diminished the severity of colonic damage in the HFD animals. That effect was accompanied by a reduction in the mesenteric adiposity and promotion of an anti-inflammatory environment through the inhibition of proinflammatory cytokines and a partial restoration of release of protective myokines, such as irisin, and plasma adiponectin content in the voluntary-exercising animals.

It is generally accepted that a sedentary lifestyle can contribute to many chronic inflammatory diseases [20]. An important role of obesity, particularly abdominal adiposity, has been suggested for the pathogenesis of various autoimmune diseases [21,22]. IBD was conventionally linked with low body weight [23,24] but a growing body of evidence for the role of abdominal adiposity and perivascular fat in the pathogenesis of IBD in these patients has been recently accumulated [25,26]. For instance, the creeping fat in patients with Crohn’s disease (CD) has been referred to as the inflammatory mesenteric fat hypertrophy mediated by a rise in proinflammatory adipokines, including TNF-α, TWEAK, leptin and IL-1β [27]. That proinflammatory activity of adipose tissue is believed to act as a prospective risk factor for increased disease activity in CD [28,29].

A pronounced role of mWAT in the pathogenesis of human IBD and experimental colitis in animal models was confirmed by clinical and experimental studies [30,31]. However, the role and a potential mechanism by which physical activity could ameliorate the course of UC and CD in humans and in experimental colitis has not been thoroughly studied yet. That is why we selected the approach of HFD feeding, which is known to induce obesity in mice, to test the hypothesis that voluntary exercise could improve the outcome of experimental colitis by affecting the expression of mucosal and adipose proinflammatory and anti-inflammatory biomarkers, including irisin released from exercising skeletal muscles. In our study, HFD feeding was associated with increased whole-body weight, a greater calorie intake and an increased percentage of relative weight of the mesenteric fat mass in the abdominal cavity in comparison with those in lean SD mice. That observation is consistent with earlier studies in which enlargement of mesenteric fat depots was observed along with an increased expression of proinflammatory cytokines in mWAT [32]. We observed increased expression of mRNA for TNF-α, MCP-1, IL-6 and leptin in HFD mice and that effect was accompanied by a rise in the plasma levels of TNF-α, MCP-1, IL-6, IL-13 and leptin. In fact, an increase in cytokines, including the ‘cytokine triumvirate’ of TNF-α, IL-6 and IL-13, has been recently proposed to play a role in the progression of experimental colitis and human IBD [33]. Previous studies revealed that UC can be considered as a Th2-dominant disease but in the last years, many studies proved that UC is characterized by a Th2 atypical immune response in which higher levels of IL-13 ha been documented [34,35]. Interestingly, that increase in the plasma IL-13 levels in our study was attenuated when the mice were subjected to voluntary exercise. A similar trend was observed when the plasma levels of IL-17, IL-1α, keratinocyte chemoattractant (KC) and IL-4 were analysed in the groups of sedentary vs. exercising HFD mice with TNBS colitis, as the initial rise in the plasma levels of these factors in the sedentary HFD mice with colitis was ameliorated by physical voluntary training. Our data is in variance with the results by Pence et al. [36] who compared cutaneous wound healing in exercised obese mice and lean mice. The authors were unable to detect any differences in gene or protein expression of IL-1β, TNF-α or IL-10, the chemokines monocyte chemoattractant protein-1 or KC in the wounds in exercised and sedentary mice [36]. However, exercise training in obese mice with a treadmill used for 60 min/day reduced macrophage infiltration and inflammatory chemokines in the adipose tissue by attenuation of the infiltration of neutrophils [37]. Our data pertaining to the increase of IL-17 plasma levels is corroborative with the observation that the expression of the Th17 cytokine IL-17 was increased in the mice model for TNBS-induced colitis [38]. Zhang et al. [39] proposed that IL-17 and its receptor IL-17R signalling play a critical role in the development of TNBS-induced colitis and may represent a target for therapeutic intervention in human IBD. Discrepancies related to the results concerning the interrelationship between obesity, exercise and healing of various wounds can be attributed to different experimental conditions and designs, in particular to studies regarding the selection of bouts of exercise, time duration of high-fat diet feeding to induce obesity and assessment of different wounds.

Previous studies documented that TNBS-induced experimental colitis resembles the changes described in CD patients which were accompanied by an increased expression of proinflammatory mediators in the mesenteric fat depots [32,40,41,42]. Our present study is in keeping with the previous findings on leptin expressed in mWAT in rodents with experimental colitis and in CD patients [42,43,44,45,46,47,48,49,50,51,52]. The importance of the leptin derived from abdominal fat for the pathogenesis of colitis is supported by an observation that treatment with a leptin antagonist ameliorated the development of chronic experimental colitis [46]. We found that obese sedentary mice exhibited an increase in the plasma leptin levels but simultaneously showed a fall in the plasma adiponectin levels as compared to the animals on a standard diet. Interestingly, the plasma levels of adiponectin and that adipokine’s expression in mWAT were significantly decreased in the mice with TNBS-induced colitis and that decrease, reflecting the status of adiponectin in our study, was more pronounced in the animals fed a HFD. Adiponectin, which is inversely affected by obesity and shows a structure similar to TNF-α, has been proposed to antagonize that proinflammatory cytokine by competing with the TNF-α receptor [53,54,55,56]. There are conflicting results related to the circulating levels of adiponectin in patients with IBD [48,50,54,57,58,59,60,61]. However, recent studies demonstrated that lower levels of serum and mesenteric adiponectin in active CD patients clearly suggest a defective regulation of that anti-inflammatory adipokine’s pathway in the pathogenesis of CD [53]. Sideri et al. [32] have shown an increase in the adiponectin mRNA expression in the mesenteric depots but the adiponectin mRNA expression in the mesenteric adipose tissue was markedly decreased in the mice fed a HFD following TNBS administration. Physical activity was reported to exert beneficial health effects by inducing anti-inflammatory actions, which was aligned with our results [62]. High intensity endurance exercise led to systemic inflammation and immunosuppression but in contrast, a regular but moderate physical activity had an anti-inflammatory effect [63,64]. Recently, we have reported that the plasma level of adiponectin was significantly decreased in rats with experimental colitis fed a HFD and that was partly reversed by forced treadmill exercise [6]. Interestingly, voluntary exercise normalized the expression of inflammatory mediators and attenuated the activation of NF-κB in rats fed a HFD [6]. In the present study, voluntary exercise reversed the fall in the plasma adiponectin levels and the expression of that adipokine in mWAT but simultaneously, the expression of the proinflammatory adipokine leptin was downregulated in the mice fed a HFD. Liu et al. [7,65] have reported an increased expression of inflammatory mediators in the colons of sedentary mice fed a HFD, an effect mediated by a decrease in the expression and activity of protective PPAR-γ.

These protective effects of voluntary exercise, as reflected by attenuation of the expression and activity of proinflammatory biomarkers in experimental colitis, could also be mediated by the substances called ‘myokines’ which are released from the skeletal muscle in response to physical training and muscle shiver [12,62,66,67,68,69]. In IBD, myokines such as irisin may balance and counteract the effects of proinflammatory adipokines released by a pathologically modified mWAT, thus contributing to the cross-talk between the skeletal muscle and adipose tissue [5,6,67]. Irisin can have a possible therapeutic role in the course of human metabolic disease, obesity and other disorders in which exercise has been proposed as beneficial [70,71]. In our study, the plasma irisin concentration tended to increase but was not significantly altered by voluntary exercise in HFD mice without colitis. That effect was reversed in animals subjected to voluntary exercise, which supported our notion that irisin could be involved in the mechanism of exercise-induced improvement in intestinal resistance to damage and the healing in experimental colitis. In contrast, a pronounced increase in the plasma irisin levels was reported by our group in rats without colitis fed a HFD and forced to perform treadmill exercise (6). Such discrepancies between the results regarding irisin in our present study and previous findings (6) can be explained not only by the difference in species (rats vs. mice), but also by different types and durations of exercise performed by the rodents in the present work (voluntary exercise by wheel running) and our previous study (forced endurance training using a two-lane treadmill). It is of interest that mWAT can also act as a source of FNDC5, whose expression is upregulated after exercise [72,73,74,75]. It is also interesting that mice with TNBS-induced colitis had a significantly lower FNDC5 expression in mWAT and such an effect was intensified in HFD animals, which supports the involvement of irisin in the cross-talk between the skeletal muscle and adipose tissue. Indeed, obese human subjects with IBD exhibited downregulation of FNDC5 gene expression in the muscle and adipose tissue along with enhanced release of proinflammatory cytokines being possibly mediated by substance P [74,76,77]. The results of our study indicating an elevation in the proinflammatory cytokine TNF-α, MCP-1 and enhanced IL-6 expression coincide with the observation that significantly higher plasma TNF-α levels, an increased DAI, formation of a fistula and a greater number of extraintestinal manifestations were present in CD patients. All these, however, appear unrelated to the plasma histamine concentrations in those patients [77].

## 5. Conclusions

We have documented that healing in experimental colitis is impaired in mice fed a HFD and that such an effect is accompanied by a decrease in the colonic microcirculation and adiponectin concentration in intestinal circulation and by an increase in plasma proinflammatory biomarkers in the mesenteric fat. Ingestion of HFD caused a pronounced change in the mesenteric WAT, indicating in particular that mesenteric fat can be an important pathogenic factor in experimental colitis affecting the macroscopic, microscopic and functional alterations over the course of colitis. Voluntary physical activity can improve colitis by diminishing the severity of colonic damage due to an increase in the colonic blood flow which is linked to protective myokines, such as irisin, released from the skeletal muscle, a phenomenon especially observed in exercising HFD mice. Our study implies that regular voluntary exercise could improve the course of IBD in obese patients due to reduction of mesenteric WAT depots, release of irisin, restoration of protective adiponectin and promotion of an anti-inflammatory environment by inhibition of proinflammatory cytokines.
